# Use of Sildenafil in young adults: a growing health problem

**DOI:** 10.11604/pamj.2023.44.113.39168

**Published:** 2023-03-01

**Authors:** Mayur Wanjari, Sampada Late

**Affiliations:** 1 Department of Research and Development, Jawaharlal Nehru Medical College, Datta Meghe Institute of Medical Sciences, Sawangi, Wardha, Maharashtra, India,; 2 Department of Nursing, Government Hospital Samudrapur, Wardha, Maharashtra, India

**Keywords:** Sildenafil, young adults, erectile dysfunction, health problems

## To the editors of the Pan African Medical Journal

We are writing to bring attention to a concerning issue affecting young adults and their health, the use of Sildenafil, commonly known as Viagra, without medical supervision. This problem has been growing rapidly in recent years, and we must take action to address it. The Food and Drug Administration (FDA) approved Sildenafil in 1998 to treat erectile dysfunction in men. The drug works by increasing blood flow to the penis, allowing for an erection to occur. However, despite its limited approved use, Sildenafil has become a popular recreational drug among young adults without a medical need. This is a dangerous trend that poses significant risks to the health and well-being of young adults [1]. We reported the case of a 26-year-old male to the emergency department with a headache, skin flushing, indigestion, and visual disturbances for 1 hour. On the physical examination and history taken, the patient revealed he took two tablets of Sildenafil to increase the duration of the sexual intercourse. The patient was advised for admission under the doctor's supervision, and after 6 hours, the patient felt better and was discharged from the hospital.

The first and most immediate risk associated with using Sildenafil without medical supervision is the potential for interactions with other medications. Sildenafil can interact with other medicines in dangerous ways, causing serious side effects that can be life-threatening. For example, Sildenafil can interact with nitrates found in certain medications, such as nitroglycerin, leading to a dangerous drop in blood pressure. This can cause serious health problems, including heart attack and stroke [2].

In addition to the potential for interactions with other medications, Sildenafil can also lead to long-term health problems. Some studies have shown that the drug can cause vision loss and hearing loss in a small number of users. Additionally, the drug can cause priapism, a painful condition in which an erection lasts for several hours or more. This can cause serious health problems and be extremely painful [3]. The use of Sildenafil can also lead to psychological dependence. Young adults who use the drug recreationally may come to rely on it to achieve an erection. This can lead to further health problems and damage relationships [4].

Another concern with using Sildenafil by young adults is the lack of regulation surrounding the sale of the drug. Currently, there are no regulations to prevent young adults from purchasing drugs without a prescription. This makes it easy for young adults to access the drug, putting them at risk of serious health problems [4]. Given the numerous risks associated with the use of Sildenafil by young adults, we must take action to address this growing problem. Parents, educators, and healthcare providers can all play a role in educating young adults about the dangers of recreational prescription medications. This education should include information about potential interactions with other medicines, long-term health problems, and the risks of psychological dependence [5].

In addition to education, stricter regulations must be put in place to prevent the sale of Sildenafil to young adults without a prescription. This could include more stringent age verification processes, greater online sales monitoring, and increased enforcement of existing regulations.

## Conclusion

Using Sildenafil by young adults is a growing problem that poses significant risks to their health and well-being. By working together, we can educate young adults about the dangers of using prescription medications recreationally and put stricter regulations to prevent the sale of Sildenafil to those who do not have a medical need for the drug. By taking these steps, we can help ensure that young adults have access to safe and effective medical treatments when needed.
